# Influence of Citrates and EDTA on Oxidation and Decarboxylation of Betacyanins in Red Beet (*Beta vulgaris* L.) Betalain-Rich Extract

**DOI:** 10.3390/molecules27249054

**Published:** 2022-12-19

**Authors:** Katarzyna Sutor-Świeży, Justyna Proszek, Łukasz Popenda, Sławomir Wybraniec

**Affiliations:** 1 Faculty of Chemical Engineering and Technology, Department C-1, Cracow University of Technology, ul. Warszawska 24, 31-155 Cracow, Poland; 2 NanoBioMedical Centre, Adam Mickiewicz University, ul. Wszechnicy Piastowskiej 3, 61-614 Poznan, Poland

**Keywords:** dehydrogenation, decarboxylation, xanbetanin, neobetanin, red beet root, colorants, betanin, betalain-rich extract, decarboxy-betacyanins, dehydrogenated betacyanins

## Abstract

The influence of stabilizing activity of citric buffers on betacyanins, as well as their thermal dehydrogenation and decarboxylation in a beetroot betalain-rich extract (BRE), was studied at pH 3–8 and temperature 30, 50 and 85 °C with an additional effect of EDTA. In acetate/phosphate buffers, the highest stability is observed at pH 5 and it decreases toward pH 3 as well as pH 8, which is more remarkable at 85 °C. For the citrates, a contradictory effect was observed. Citric buffers tend to stabilize the substrate pigments and their intermediary products in acidic solutions, although increase their reactivity at pH 6–8. The highest impact of EDTA addition on pigment retention in acetate buffers is observed at 85 °C and pH 3–5 as well as 8, reflecting the preserving activity of EDTA at the most unfavorable conditions. At lower temperatures, pigment stability in more acidic conditions is still at higher levels even without addition of citrates or EDTA. The most striking effect on generation of betanin derivatives during heating is 2-decarboxylation which preferentially proceeds in the most acidic environment and this generation rate at 85 °C is much higher in the citrate buffers compared to acetates.

## 1. Introduction

Natural food colorants are employed as substitutes for synthetic dyes in the food industry due to concerns related to safety as well as an increasing desire to avoid “synthetic” ingredients. Artificial colorants in foods and dietary supplements have been increasingly questioned by consumers, and instead, natural plant pigments, such as betalains [1,2,3,4,5,6,7,8,9] are researched and studied as new, natural substances in these diverse applications. Natural food colorants, frequently possessing high antioxidant properties [10,11,12] as well as important pharmacological activities, prevent various adverse health problems such as carcinogenic, allergic and toxic effects by substitution of strong and harmful artificial reagents [13,14,15,16,17,18]. Colour sensitivity of natural colorants on changing pH and quality of foodstuffs enables colorant application as controlling markers. Additionally, natural colorants promote food functional properties as they improve the nutritional quality of foods and are sources of several essential vitamins, and promote food sensory attributes [19,20,21,22,23].

**Figure 1:**
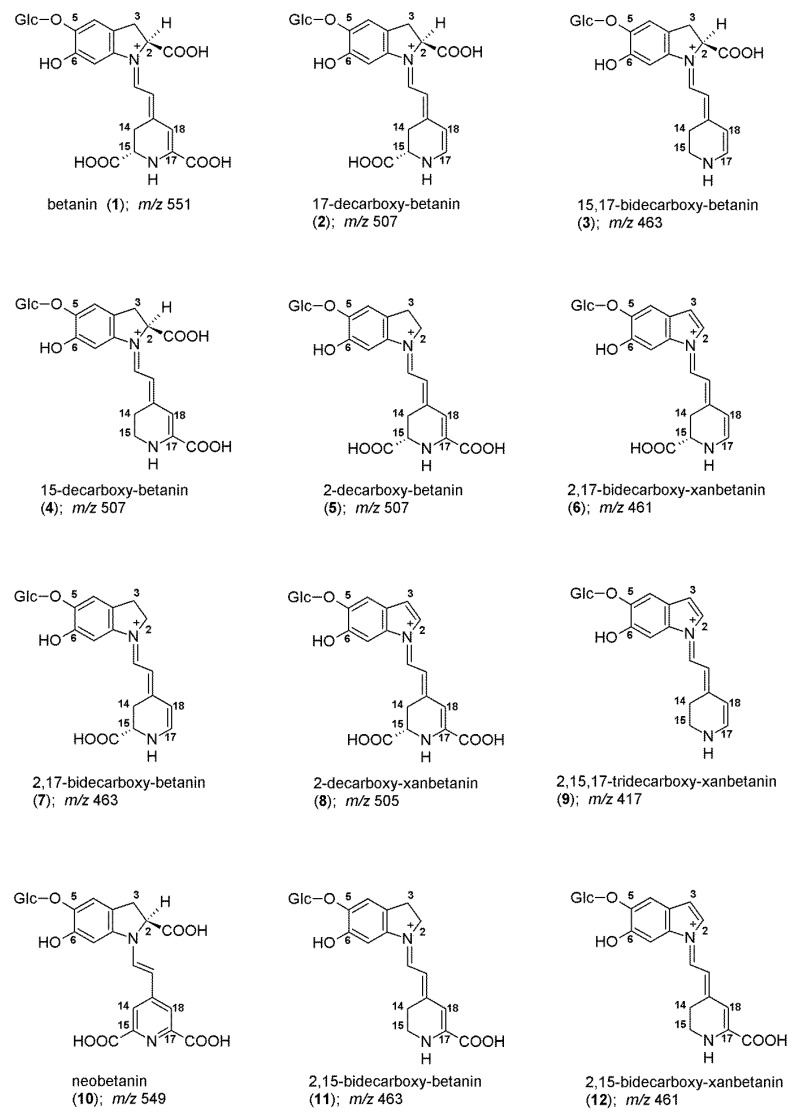

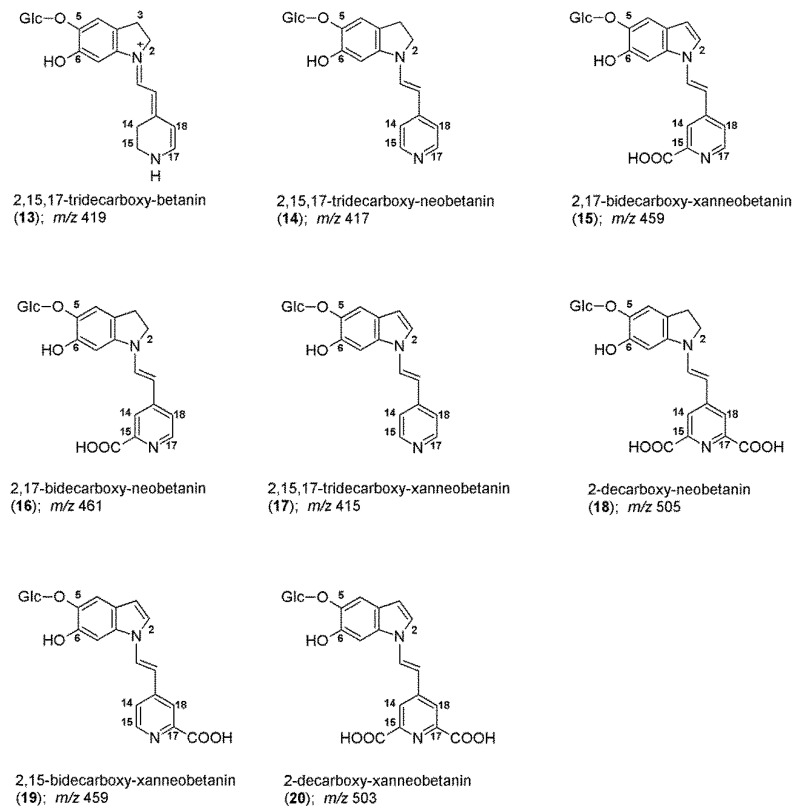
Chemical structures of betanin and its decarboxylated and dehydrogenated derivatives detected during the heating experiments of the betalain-rich extract/concentrate (BRE).

Structurally, red–violet betacyanins, such as betanin/isobetanin (Figure 1), belong to betalain family which represents a class of N-heterocyclic water-soluble plant pigments giving a wide variety of colors to flowers and fruits [1,2,3,4,5,6,9]. These pigments are biosynthesized at high quantities in red beetroot (*Beta vulgaris* L.), a widely consumed vegetable that contains significant amounts of nutritious and bioactive compounds which are also found in dietary supplements. Betanin E-162 colorant is vastly produced from red beetroot [24,25,26] and its concentrates are prepared by vacuum evaporation of beet juice, or as powders made by spray-drying of the concentrate. Many betacyanins, such as betanin, are 5-*O*-glucosides of betanidin (the basic chromophoric aglycone unit).

Use of natural pigments such as betalains in food and health-related products is often limited by said pigments’ relative oxidative stabilities in the products or physiological matrices. Recent studies on determination of betacyanin oxidation mechanism may inform future development and delivery of better stabilized molecules for improved outcomes.

The pharmacological properties of betalains are mainly related to their antioxidant activity and the prevention of tissue damage. As natural antioxidants, they are non-toxic and safe when consumed on a regular basis, and the positive consequences of their action, such as slowing down the aging processes or inhibiting the development of serious diseases, are invaluable. Therefore, they are more and more often used as substitutes for synthetic dyes in food, cosmetic and pharmaceutical products, and numerous studies are being carried out to expand their use for therapeutic purposes. Betacyanins are considered to be highly active natural compounds used as dietary supplements in such products as beetroot extracts [26] with potential benefits to human health [27,28,29,30,31,32,33,34,35,36,37]. Recent studies indicate a particularly beneficial effect of betacyanins as antioxidants in different types of pathologies associated with oxidative stress [27,28,33,34,35,36,37,38,39,40,41]. The influence of betalain-rich extract on the reduction of discomfort associated with osteoarthritis was reported [16]. There has been a growing interest on betacyanins as potential chemopreventive agents capable of stopping tumor growth, indicating that the sources of betacyanins (including the root of *B. vulgaris*) deserve increased attention in search of anticancer preparations [31,32,42,43].

Food preparations received at different industrial conditions can strongly differ in terms of the presence of degradation betalainic products, and consequently in their stability and colour properties. Stability of betalains depends on many factors such as the pH, temperature, the presence of oxygen, water and light which limit the possibilities of application of these pigments as dyes in food [44,45,46,47,48,49,50,51,52,53,54,55,56,57,58,59,60,61]. Studies on the effect of the acylation or a presence of other groups in the structure of betalains on the stability of pigments were conducted [46]. However, within the last fifteen years, the structures of the new decarboxylated and dehydrogenated derivatives were determined for betanin and acylated betacyanins [62,63,64,65,66,67].

Therefore, we see a need for performing systematic studies on the determination of transformation directions for specific betacyanins as well as their mixtures in selected matrices. Some compounds present in the food are affecting the stability of betalains. Contradictory data on determining the influence of ascorbic acid on betanin stability were a result of different experimental conditions [46,51,53]. In spite of a confirmed positive stabilizing effect of ascorbic acid on betacyanins [56], in earlier studies, instead of increased stability, a degradation of betanin was observed at higher concentrations of ascorbic acid, considering products of ascorbic acid oxidation as responsible for the decomposition [46,47,55,57]. In addition, in several reports, replacing ascorbic acid with isoascorbic acid enhanced betanin stability [53,58,59,60,61].

A significant influence on betalain stability is observed for pH. The optimal pH value for betalain stability fluctuates around 5.5 [45,46,47,48]. Ions of transition metals are lowering betanin stability during storage as well as at higher temperatures [46,54]. The presence of chelating compounds such as EDTA and citric acid reduces the degradation of betalains. The effect of 14 metal cations (Ni^2+^, Cd^2+^, Ag^+^, Co^2+^, Cu^2+^, Fe^2+^, Fe^3+^, Hg^2+^, Mn^2+^, Pb^2+^, Sn^2+^, Zn^2+^, Al^3+^ and Cr^2+^) on betanin and betanidin degradation was investigated in aqueous and organic-aqueous solutions by spectrophotometry [68]. The presence of organic solvents (methanol, ethanol and acetonitrile) affects substantially the pigments decomposition in acidic media induced by metal cations whose degrading action in such media is significantly higher than in aqueous solutions [55,68].

The influence of Cu^2+^ on the degradation was studied in a more detailed manner because of its ability to form complexes with betanin. Formation of the complexes is followed by a fast degradation of the pigments. Acidification of the solutions restores the free pigments. This reflects the competitive action of hydrogen ions leading to the lower dissociation of two carboxylic groups of the pigment (at the C-15 and C-17 carbons) which presumably take the part in the complexation process, together with the electron pair donating N-16 nitrogen [68]. The overall effect can be reversed by an addition of EDTA as well as other potent chelating agents. Recent research confirmed that Cu^2+^-catalyzed oxidation of betanin and 2-decarboxy-betanin results in the generation of neo-derivatives of betanin [66]. In contrast, Cu^2+^-catalyzed oxidation of 17-decarboxy-betanin and 2,17-bidecarboxy-betanin results mostly in formation of betanin xan-derivatives. A relevant mechanism of Cu^2+^-catalyzed oxidation of the pigments was proposed suggesting that the oxidation of betanin can possibly occur in the region of the dihydropyridinic ring and can omit the stage of methide quinone formation in the dihydroindolic system.

Citric acid was found to improve betacyanin stability [54,57,69], though being less effective than ascorbic and isoascorbic acids [57]. Recently, extraction of betalains was evaluated using different aqueous and organic solvents and then integrated into the purification process using the aqueous two-phase systems (ATPS) with extensive application of the containing citrate buffers [70]. In our laboratory, however, the use of citrates was frequently associated with unpredictable changes in betalain integrity, especially at elevated temperatures, therefore, the influence of citrate buffers on red beet betacyanin stability and reactivity has been explored in this contribution.

Recently, the first qualitative results on thermal oxidation (resulting in dehydrogenation) interrelated with decarboxylation of betacyanins present in a specifically purified highly concentrated betalain-rich extract (BRE) in selected acetate/phosphate buffers were presented, which are of particular significance for formulation and performance of foods [71]. Based on that report, a closer insight into the mechanism of betacyanin degradation was presented, complementary to the previous discussions presented in our publications.

In this contribution, the influence of two stabilizing agents, EDTA and citric buffers, which change the access to traces of metal cations on stability of betacyanins as well as their thermal dehydrogenation and decarboxylation at a temperature range of 30–85 °C was investigated by spectrophotometric monitoring of the beetroot betalain-rich extract heating reaction progress as well as liquid chromatographic and mass spectrometric analyses, which may be of particular significance for formulation and performance of foods.

## 2. Results and Discussion

### 2.1. Spectrophotometric Monitoring of the BRE Heating Reaction Progress

For the aim of establishing a basic experimental platform in the following sections for a more comprehensive discussion of the influence of citrates/EDTA on generation/degradation of selected species during heating, we prepared the reaction mixtures and initially monitored the reaction progress by spectrophotometry. The highest temperature tested was established at 85 °C in order to conform to previous preliminary studies performed at the same temperature [46,56,71,72], which is high enough to perform relatively fast but convenient to control processes within a 1 h time frame. This is also of great importance for the preparation of real compositions based on betalain-rich extracts depending on requirements concerning such end-products as functional foods. In fact, there is still a lack of knowledge of bioactivity and reactivity of betanin derivatives which still possess the betalainic chromophoric system, therefore, this report shall initiate a basic discussion on possible pro-health applications of processed betalain-rich extracts. Performed experiments were expanded to lower temperatures which enable controlling pigment reactions at less drastic conditions over a longer time (50 °C) or are compatible with unfavorable conditions during food storage (30 °C). Such extensive studies on EDTA and especially citrate influence on a vast group of betalainic derivatives are presented for the first time.

**Figure 2:**
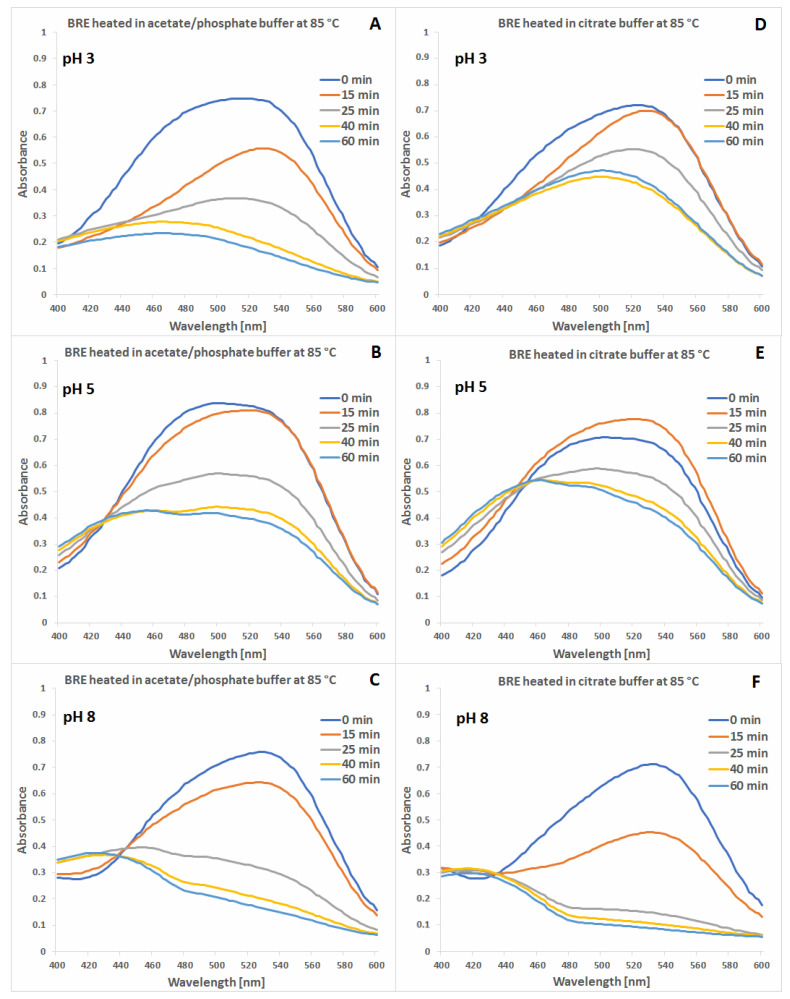
Visible spectra of betalain-rich extract (BRE) samples collected during their heat processing at 85 °C in acetate/phosphate (**A**–**C**) and citrate buffers (**D**–**F**) at pH 3, 5 and 8.

**Figure 3:**
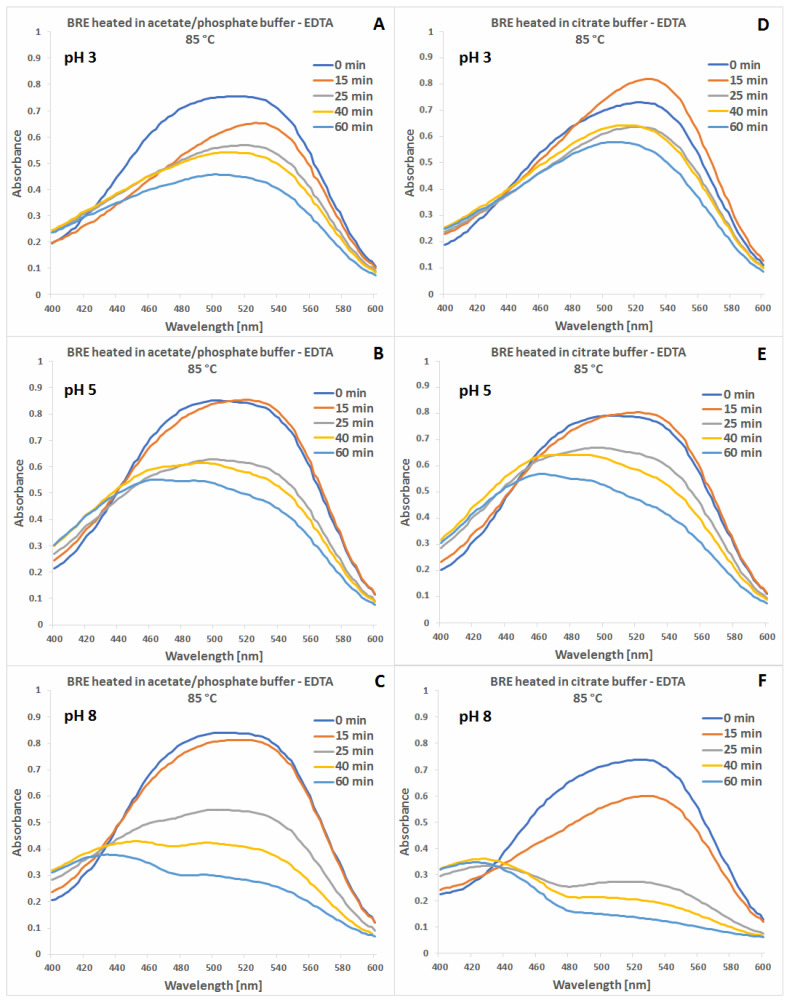
Visible spectra of betalain-rich extract (BRE) samples containing EDTA collected during their heat processing at 85 °C in acetate/phosphate (**A**–**C**) and citrate buffers (**D**–**F**) at pH 3, 5 and 8.

The spectra of the betalain-rich extract samples collected in the visible range during heat processing in acetate/phosphate and citrate buffers at 85 °C are shown in Figure 2 and Figure 3. A 1 h processing time range established for 85 °C was based on the reaction progress in the acetate buffer at pH 3 resulting in almost complete flattening of the spectrum at the end (Figure 2A). Taking this sample’s time-dependent profile as a reference, it can be stated that there are noticeable differences between the solutions processed in both types of buffers.

The absorption maxima levels as well as their shifting during the heating process at pH 3–5 and 85 °C suggest that citric buffers tend to stabilize not only the substrate pigments but also their intermediary products which are listed in the following sections (Section 2.2, Section 2.3 and Section 2.4). In addition, in this pH range, addition of EDTA has the highest impact on increasing stability of the pigments (Figure 3).

The group of generated dehydrogenated pigments absorbs light mainly in the range of 400–480 nm, therefore, following slower changes of the spectra in the range of 400–480 nm in citric buffers at pH 3–5 than in the acetate-phosphoric buffers enables explanation that the citrates retard the compound decomposition during heating. Similar trend is observed for the group of decarboxylated betacyanins together with the natural betacyanins (Table 1) in the spectra range of 500–540 nm.

**Figure 4:**
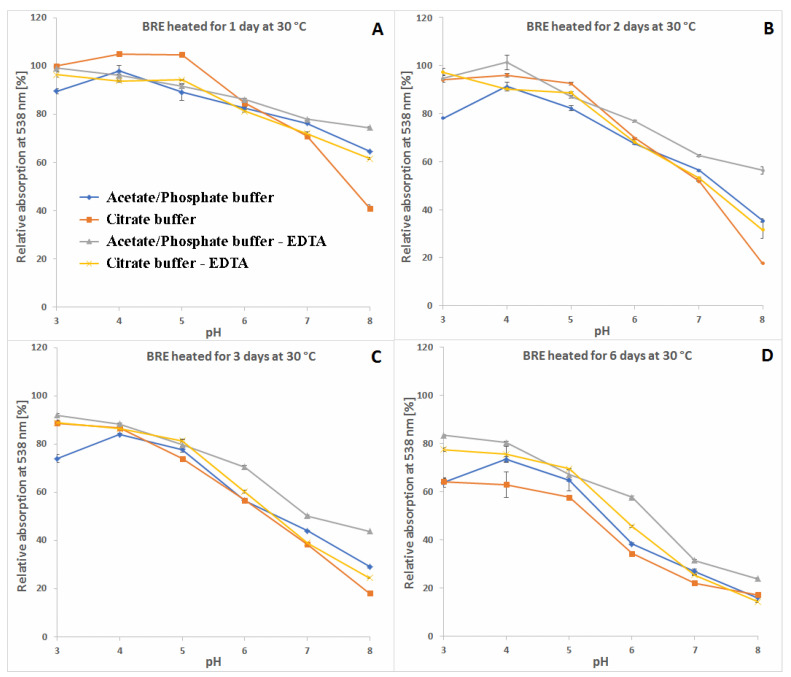
Relative absorption of red-violet pigments monitored at λ 538 nm after 1 (**A**), 2 (**B**), 3 (**C**) and 6 days (**D**) of heating (30 °C) of betalain-rich extract (related to its starting value before heating) in acetate/phosphate and citrate buffers with optional addition of EDTA.

**Figure 5:**
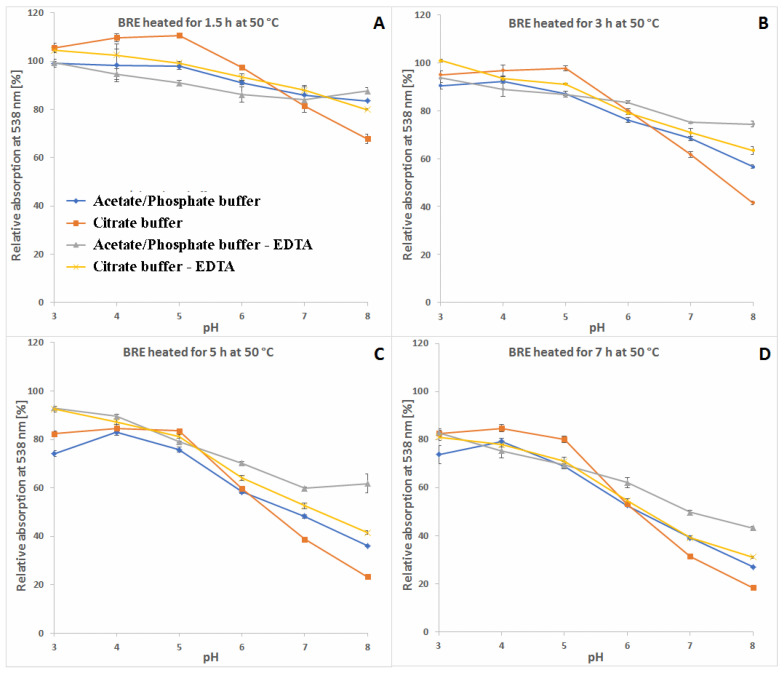
Relative absorption of red-violet pigments monitored at λ 538 nm after 1.5 (**A**), 3 (**B**), 5 (**C**) and 7 h (**D**) of heating (50 °C) of betalain-rich extract (related to its starting value before heating) in acetate/phosphate and citrate buffers with optional addition of EDTA.

**Figure 6:**
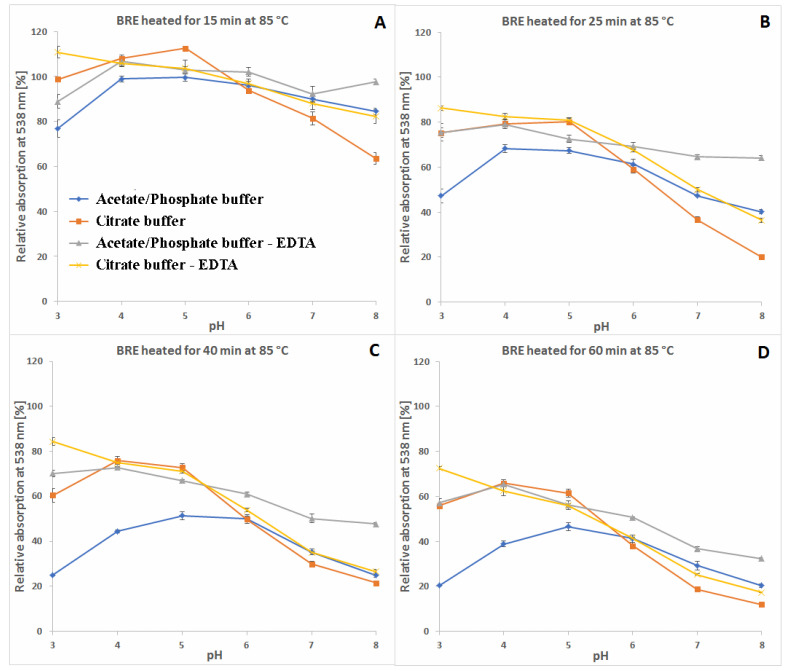
Relative absorption of red-violet pigments monitored at λ 538 nm after 15 (**A**), 25 (**B**), 40 (**C**) and 60 min (**D**) of heating (85 °C) of betalain-rich extract (related to its starting value before heating) in acetate/phosphate and citrate buffers with optional addition of EDTA.

A more comprehensive view of the influence of pH and buffer constituents on absorption at the characteristic wavelength of 538 nm for betacyanins, monitored during heating, related to its starting value [%] at temperatures 30, 50 and 85 °C is presented in Figure 4, Figure 5 and Figure 6. In general, in acetate/phosphate buffers, the highest stability is observed at pH 5 and it decreases toward pH 3 as well as 8, which is more remarkable at 85 °C. At lower temperatures, the relative absorption in more acidic conditions is still high, even without the addition of citrates or EDTA. This suggests that applying low pH is more destructive for the pigments only at high temperatures.

Furthermore, the highest impact of EDTA addition on relative absorption in acetate buffers is observed at 85 °C and pH 3–5 as well as 8, reflecting the preserving activity of EDTA at the most unfavorable conditions. In the case of applied citrate buffer, the relative absorption is much higher than in the acetate buffer (pH 3–5) and the addition of EDTA increases it only by a small value, which is negligible at pH 4–6 (Figure 6). In general, the influence of EDTA and citrates is not as remarkable at lower temperatures, 30 and 50 °C (Figure 4 and Figure 5), as at 85 °C (Figure 6).

As mentioned above, the addition of citric acid increases the absorption of the heated mixtures which is best observed at pH 3–5 and 85 °C. At the initial period (1.5 h and 15 min) at 50 and 85 °C, respectively, the relative absorption reaches the level above 110% (Figure 5A and Figure 6A) which is also assisted by addition of EDTA. This can be a result of a tentative generation of red–violet decarboxylated betanin derivatives **4**, **5/5′** and **11** (Table 1) which may have higher extinction coefficients than betanin **1**, hence at the beginning, their initial generation increases light absorption in comparison to the starting mixture.

**Figure 7:**
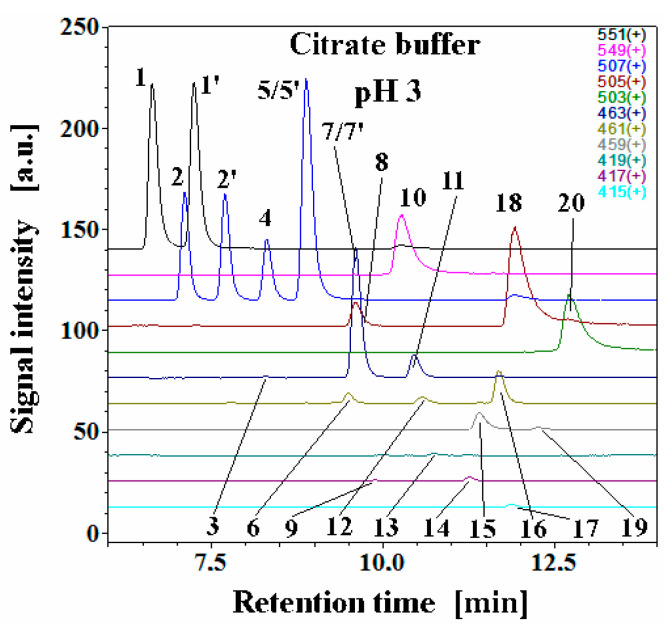
Chromatographic LC-MS profiles of selected ions of betanin as well as its decarboxylated and dehydrogenated derivatives generated in betalain-rich extract after 60-min heating experiments in citrate buffers at pH 3 and 85 °C.

**Figure 8:**
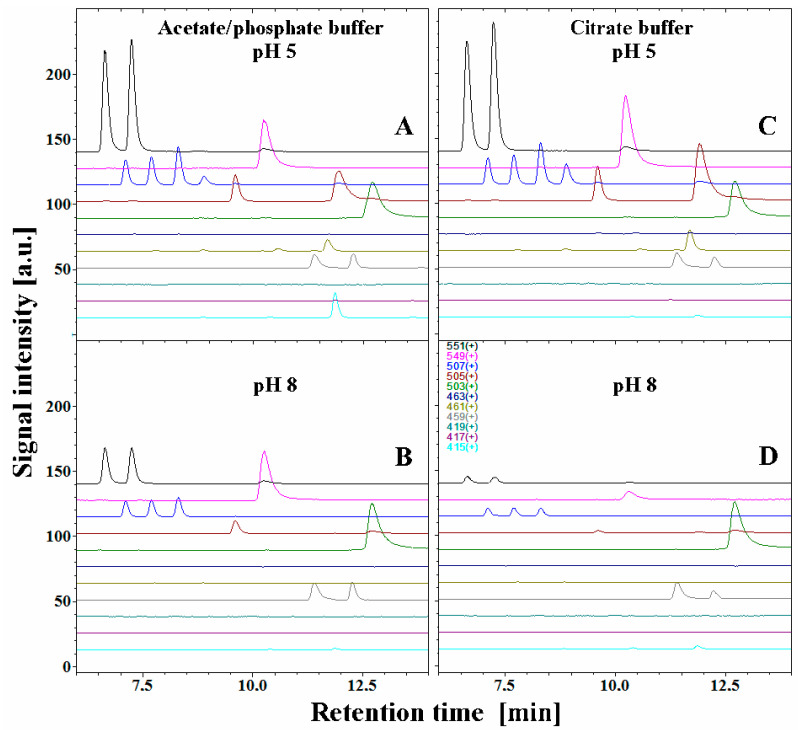
Chromatographic LC-MS profiles of selected ions of betanin as well as its decarboxylated and dehydrogenated derivatives generated in betalain-rich extract after 60-min heating experiments in acetate/phosphate (**A**,**B**) and citrate buffers (**C**,**D**) at 85 °C—pH 5 and 8.

In contrast to acidic solutions, at pH 8, the citrate buffers diminish the relative absorption of the heated mixtures during the whole process and all the temperatures (Figure 4, Figure 5 and Figure 6), promoting additional reactions which is also reflected in obtained LC-MS chromatograms (Figure 7 and Figure 8) with smaller number of chromatographic peaks.

### 2.2. Chromatographic and Mass Spectrometric Monitoring of the Products Generated during the BRE Heating Experiments under the Influence of the Citric acid Concentration

The exemplary chromatographic profiles of detected betanin decarboxylated and dehydrogenated derivatives (Figure 1) obtained during the heating experiments of the betalain-rich extract/concentrate (BRE) in acetate/phosphate and citrate buffers at 85 °C are presented in Figure 7 and Figure 8. The monitoring of this huge group of betalainic derivatives as well as comprehensive discussion of the influence of selected factors on the levels of these compounds in the resulting products is presented for the first time.

For this aim, recently, we performed identification of known as well as novel betanin derivatives by LC-DAD-MS during preliminary experiments performed only in acetate/phosphate buffers at 85 °C (Table 1) [71] in part based on a series of already known decarboxylated and dehydrogenated betanin standards [52,62,63,64,65,66], but also based on NMR structural identification of novel decarboxylated betanins [71].

The chromatograms obtained for pH 5, which promote the highest stability of the extract, resemble a starting betalainic profile determined in a previous research [65].

The principal pigments present in the starting BRE extract are betanin **1** and its isoform **1′** as well as their main oxidation product, neobetanin **10**. The other betanin derivatives are 17-decarboxy-betanin/-isobetanin **2/2′**, 15-decarboxy-betanin **4**, 2-decarboxy-betanin/-isobetanin **5/5′**, 2-decarboxy-xanbetanin **8** and the most hydrophobic derivative, 2-decarboxy-xanneobetanin **20** (Table 1). The other derivatives, however, are also more hydrophobic than betanin/isobetanin **1/1′**. Similar chromatograms (data not shown) were obtained in the EDTA-fortified solutions (Figure 4, Figure 5 and Figure 6).

Apart from the very well-known 2,17-bidecarboxy-betanin/-isobetanin **7/7′**, two less abundant isomeric bidecarboxylated betanin derivatives **3** and **11,** which showed the maximum absorption at λ_max_ 494 and 532 nm, respectively, were also detected in BRE [67]. These compounds were assigned tentatively as 15,17-bidecarboxybetanin **3** and 2,15-bidecarboxy-betanin **11** based on analogous differences in retention times between **2** and **5** (2-decarboxylated betacyanins are eluted from the column later than 17-decarboxylated derivatives) [67]. The lack of a carboxyl group on the C-15 carbon implies the lack of chirality in this position, therefore, only single forms of the dyes are detected in the chromatograms, which additionally confirms their identification. Further recent NMR and high-resolution mass spectrometric analyses confirmed the chemical structures of 15-bidecarboxy-betanin **4** and 2,15-bidecarboxy-betanin **11** [71] which can be present at higher quantities in processed *B. vulgaris* juices and extracts [67]. This supported the proposal of occurring additional reactions accompanied by the 2,15-decarboxylation processes at different dehydrogenation levels with identified 15-decarboxy-betanin and 2,15-bidecarboxy-betanin as the distinct indicators of this route type [71].

The other oxidized derivatives, 2-decarboxy-neobetanin **18** and 2,17-bidecarboxy-neobetanin **16**, were detected previously in the BRE extract [65].

Following the recent studies [71], the other pigments were also acknowledged, such as the isomeric 2,17-bidecarboxy-xanbetanin **6** and 2,15-bidecarboxy-xanbetanin **12,** as well as doubly oxidized structures of 2,17-bidecarboxy-xanneobetanin **15** and 2,15-bidecarboxy-xanneobetanin **19.** The completely decarboxylated derivative, 2,15,17-tridecarboxy-xanneobetanin **17**, was also detected as well as small amounts of 2,15,17-tridecarboxy-xanbetanin **9**, 2,15,17-tridecarboxy-betanin **13,** 2,15,17-tridecarboxy-neobetanin **14** (Table 1).

### 2.3. Determination of Citric Acid and EDTA Influence on Betanin/Isobetanin ***1/1′*** and Neobetanin ***10*** Substrate Retention Levels during the Heating of the BRE Extract

**Figure 9:**
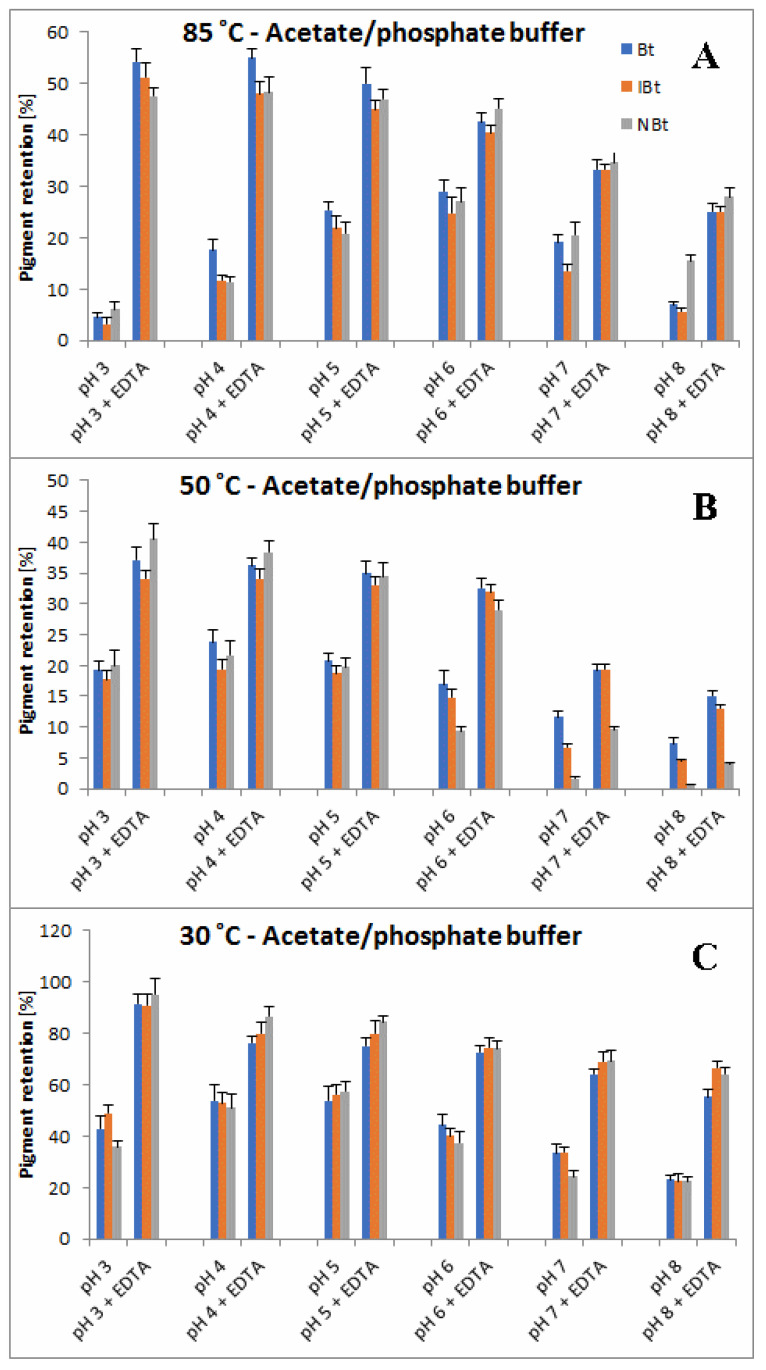
Pigment retention values [%] of betanin **1**, isobetanin **1′** and neobetanin **10** with optional addition of EDTA after long-term heating of the BRE extract for 1 (**A**), 7 (**B**) and 72 h (**C**) in acetate/phosphate at 85, 50 and 30 °C, respectively.

**Figure 10:**
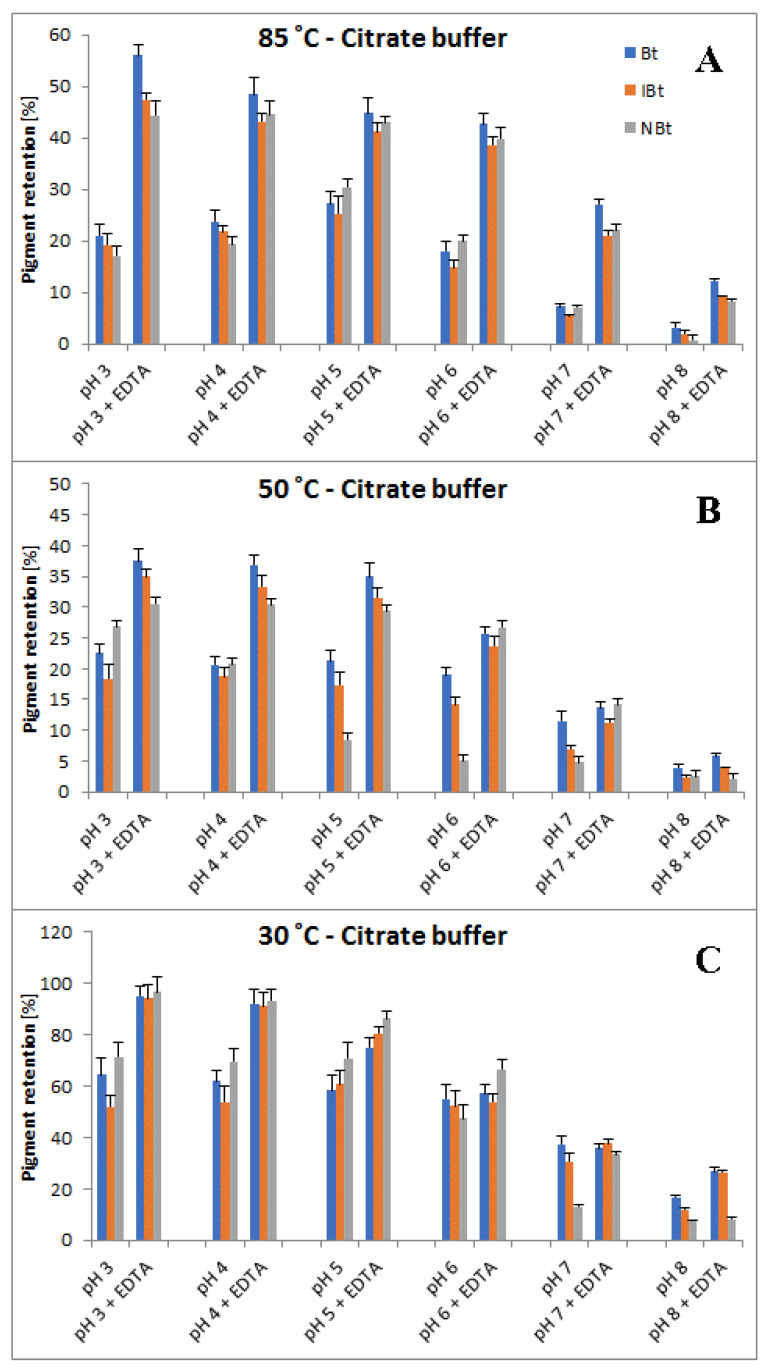
Pigment retention values [%] of betanin **1**, isobetanin **1′** and neobetanin **10** with optional addition of EDTA after long-term heating of the BRE extract for 1 (**A**), 7 (**B**) and 72 h (**C**) in citrate buffers at 85, 50 and 30 °C, respectively.

Based on the first preliminary reports on detection of neobetanin during heating of betanin preparations [46], we paid special attention to this partially oxidized pigment and, in this contribution, present completely new exploration results of the influence of citric acid and EDTA presence on the levels of neobetanin **10** in comparison to betanin/isobetanin **1/1′** in the heated solutions. Pigment retention values (calculated as ratios of the signal values obtained for the pigments after a heating period and the corresponding signals before heating) for betanin **1**, isobetanin **1′** and neobetanin **10** after long-term heating of the BRE extract in both the buffers are presented in Figure 9 and Figure 10 for temp. 30 (72 h), 50 (7 h) and 85 °C (1 h). In general, retention values obtained for these different pigments are similar and there is a significant influence of temperature and pH on retention changes observed. At 85 °C and without the addition of EDTA, the chemical transformation rate is the highest at pH 3 and 8. This is especially inferred from a very low pigment retention at pH 3 (3–6%) without EDTA in comparison to a much higher retention (47–54%) in the presence of EDTA (Figure 9A). At pH 8, betanin/isobetanin retention reaches only 5–7% without EDTA vs. 25% in the presence of EDTA. At lower temperatures (Figure 9B,C), the pigment stabilities at pH 3 are relatively much higher and similar in the pH range 3–6, decreasing at pH 7–8, confirming the spectrophotometric results (Figure 2, Figure 3, Figure 4, Figure 5 and Figure 6).

In all the acetate/phosphate buffers, addition of EDTA increases the retention of the pigments and its most striking effect at 85 °C is obtained for pH 3–4 (Figure 9A). In citrate buffers, the highest stabilizing effect of EDTA at 85 °C is also noticed for pH 3 as well as for pH 7–8 (Figure 10A). Furthermore, at 30 °C, addition of EDTA to the citrate solutions is much less effective, especially at pH 5–6 (Figure 10C), however, this is a result of high stabilizing effects of the citrates themselves.

In comparison to acetic buffers without EDTA, addition of citric buffers at 85 °C results in significant stabilizing of the substrate pigments at a retention of 17–24% (betanin/isobetanin **1**/**1′** and neobetanin **10**) at pH 3–4 (compared to 3–6% in acetic buffers) but increase their reactivity at pH 6–8 (Figure 10A) which is reflected in a lowered retention level (to 16–18% and 1–3% at pH 6 and 8, respectively). A similar tendency is observed at 30 and 50 °C for pH 7–8 (Figure 10B,C).

### 2.4. Determination of Citric Acid Influence on Signal Profiles of Chromophoric Betanin Derivatives Formed during BRE Heating

In the previous reports [56,62,67,71], results of the first studies on influence of pH on generation of chromophoric derivatives from betanin, still possesing the characteristic betalainic pattern based on the 1,7-diazaheptamethinum backbone, were presented. Such derivatives may exhibit interesting bioactive properties which can be moderated by changing their level of decarboxylation/dehydrogenation. However, no qualitative and quantitative effect of citrates on generation of a huge group of betanin derivatives **2**–**20** was explored.

Previously, the only studied effect was that of acetate and phosphate buffer pH on generation of decarboxylated betanins in heated BRE extract at 85 °C [67,71]. The most distinctive trend was observed for 2-decarboxylation taking place at pH 3–4 at the highest extent [71] with a steady generation of **2** and **4** from betanin in the whole tested pH range with the highest concentrations detected at pH 6.

**Figure 11:**
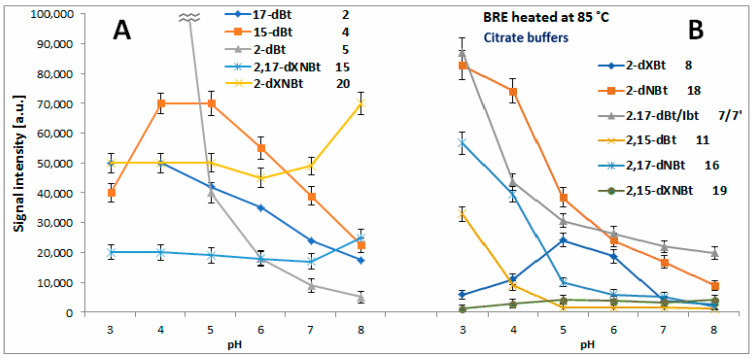
LC-MS signal levels of prominent decarboxylated/dehydrogenated betanin derivatives detected after 45 min extract heating at 85 °C in citrate buffer solutions in dependence on pH. ((**A**): 2, 4, 5, 15 and 20; (**B**): 8, 18, 7/7’ 11 16 and 19).

**Figure 12:**
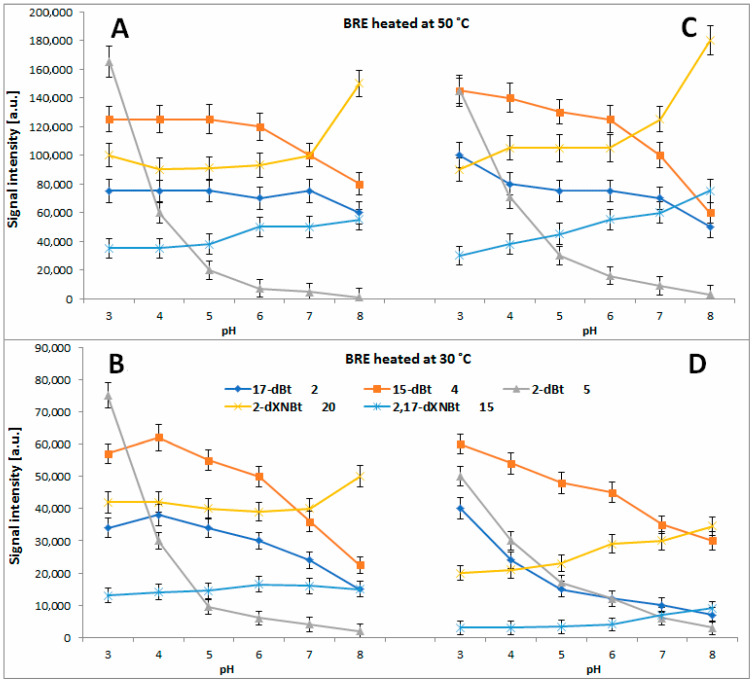
LC-MS signal levels of prominent mono-decarboxylated betanin derivatives (**2**, **4** and **5**) and most hydrophobic xanneobetanins (**15** and **20**) detected after 5 h (**A**,**C**) and 3 days (**B**,**D**) extract heating at 50 and 30 °C, respectively, in acetate/phosphate (**A**,**B**) and citrate (**C**,**D**) buffer solutions in dependence on pH.

In Figure 11A and Figure 12A–D, the chromatographic signals detected for the formed mono-decarboxylated betanins **2**, **4** and **5/5′** after 45 min, 5 h and 72 h extract heating at 30, 50 and 85 °C, respectively, are presented, compared to [71]. In the citric buffers at 85 °C, the maximum of 15-dBt **4** generation is shifted to pH 4–5 but the signals detected for 17-dBt **2** diminish strongly with increasing pH. The latter trend is also observed for **2** and **4** in the citric buffers at 30 and 50 °C (Figure 12C,D) in contrast to the non-citric buffers where the changes of the obtained signals are smaller (Figure 12A,B).

In all the tested solutions, 2-dBt/2dIBt **5/5′** is preferentially formed in the most acidic environment (Figure 11A and Figure 12A–D) and this generation rate at 85 °C is much higher in the citrate buffers compared to [71]. The level of acetic acid as well as the starting substrate, betanin/isobetanin **1/1′**, but also the presence of ethanol appeared influencing the progress of 2-dBt/2-dIBt **5/5′** formation in the previous studies [62,67]. In the current study performed on preliminarily purified BRE extract, the high acidity of the tested solutions is the main factor determining the generation of high quantities of 2-dBt/2dIBt **5/5′**.

**Figure 13:**
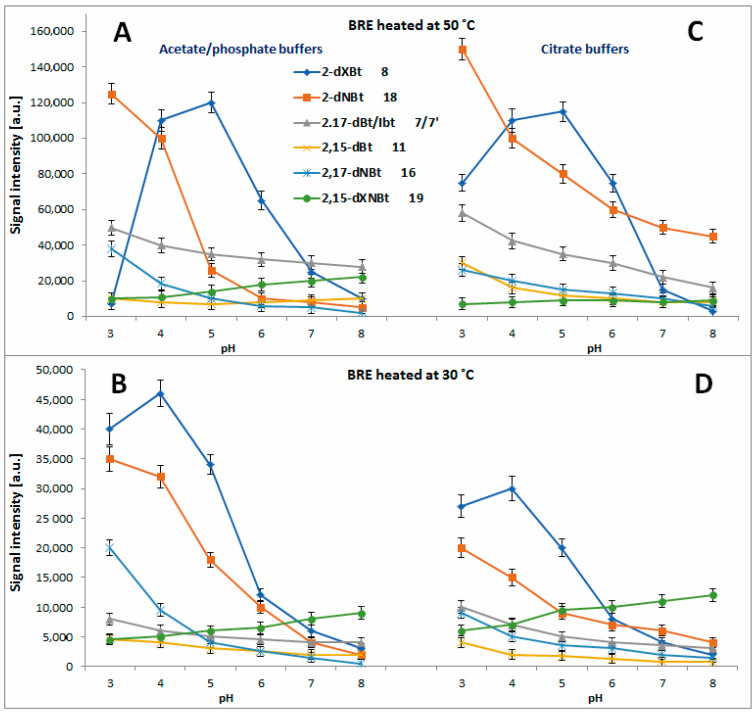
LC-MS signal levels of prominent bi-decarboxylated betanin derivatives (**7/7′** and **11**) and dehydrogenated betanins (**8**, **16**, **18**, and **19**) detected after 5 h (**A**,**C**) and 3 days (**B**,**D**) extract heating at 50 and 30 °C, respectively, in acetate/phosphate (**A**,**B**) and citrate (**C**,**D**) buffer solutions in dependence on pH.

In the next reaction steps from the intermediary mono-decarboxylated betanins, formation of two derivatives, 2,17-bidecarboxy-betanin/-isobetanin **7/7′** and 2,15-bidecarboxy-betanin **11**, follows a similar trend to **5/5′**, especially at 85 °C (Figure 11B) where the acidic environment is preferential. Even if the quantities are not as high is in the case of **5/5′**, the citrates enhance their formation at 30 and 50 °C (Figure 13A–D).

**Figure 14:**
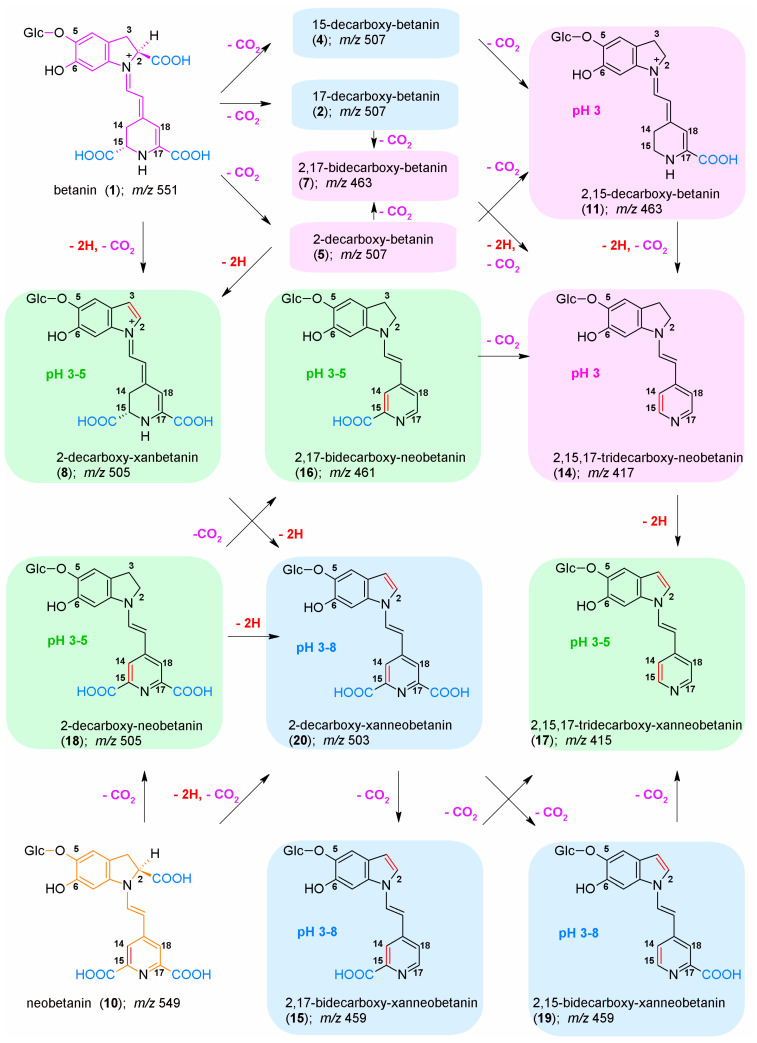
The main decarboxylation (**-CO_2_**) and oxidation (**-2H**) pathways from betanin and neobetanin occurring during the BRE heating leading to observable distinct reaction products at different pH ranges (3 (pink background); 3–5 (green background) and 3–8 (blue background)) based on LC-MS monitoring of the reaction mixtures in acetate/phosphate and citrate buffer solutions as well as on the results presented in [71].

The elucidated pattern of pH influence on betanin derivative formation during the heating of the BRE extract (Figure 14), based on the experiments in acetate/phosphate and citrate buffer solutions in this report as well as on the results presented in [71] reflects two main pathways with initial 2,3-dehydrogenation of the main extract constituents: betanin and neobetanin.

The first path follows the generation of 2-decarboxy-xanbetanin **8** from betanin **1**. This compound was observed at the highest concentration in the weak acidic environment in acetate phosphate buffer (pH 6) at 85 °C [71] and in the current study, in the citrate buffer, the optimal pH range is shifted to around 5. Additional tracing its signal levels at 30 and 50 °C enables conclusion that at high temperatures, 2-decarboxy-xanbetanin **8** is quite stable only in the pH range known as the most inert for betacyanins (pH 5–6), otherwise it undergoes further transformations (Figure 14) into more stable derivatives. Diminishing temperature to 50 °C enables higher stability of **8** which is detected at ca. two times higher level in both the buffer types (Figure 13A,C). Monitoring the presence of the derivative **8** at 30 °C (Figure 13B,D) reveals that it is generated preferably at pH 3–4, albeit at lower rate, and can exist in the solutions at low temperature even in acidic environment. Elevation of temperature increases generation but at the same time enhances further transformations of this labile intermediary product, especially in acids.

The second path proceeds by 2,3-dehydrogenation of neobetanin **10** leading to a formation of 2-decarboxy-xanneobetanin **20** [46,62,65,71] which is detected at the highest concentration levels at pH 8 as another prominent product of betanin/neobetanin degradation (Figure 11A and Figure 12A–D). This doubly oxidized pigment can be also formed from the reactive product of betanin oxidation, 2-decarboxy-xanbetanin **8**, through 14,15-dehydrogenation (Figure 14).

Another derivative, 2-decarboxy-neobetanin **18**, can be evidently generated from neobetanin **10** (Figure 14), especially at pH 3–4, by decarboxylation at carbon C-2. This compound is observed at high amounts in all the tested buffered solutions at 30, 50 and 85 °C (Figure 13). Further decarboxylation results in a formation of 2,17-bidecarboxy-neobetanin **16**, especially at high levels at elevated temperature (85 °C) and pH 3–4 in both the buffer types.

Interestingly, generation of **16** from betanin by 2-decarboxylation with concurrent 14,15-dehydrogenation is much less plausible because 2-decarboxylation is mostly accompanied by 2,3-dehydrogenation [63,64,66]. Instead, formation of 2,17-decarboxy-xanbetanin **6** and 2,15-bidecarboxy-xanbetanin **12** should be rather expected from formed 17-decarboxy-betanin **2** and 15-decarboxy-betanin **4**, respectively; however, the observed chromatographic signals for **6** and **12** are not meaningful (Figure 7) compared to [71].

A pair of derivatives detected at medium concentrations includes 2,17-decarboxy-xanneobetanin **15** (Figure 11A and Figure 12A–D) and 2,15-bidecarboxy- xanneobetanin **19** (Figure 13), which are observed at all applied temperatures and at slightly higher levels at pH 7–8.

Similar results, with lower signal values detected for the obtained reaction products (data not shown), were obtained in the EDTA-fortified solutions (Figure 4, Figure 5 and Figure 6). These compounds can be a result of decarboxylation of **20** (Figure 14) and most probably transform to the final chromophoric derivative, 2,15,17-tridecarboxy-xanneobetanin **17** observed at low concentrations, mainly in acetic buffers at pH 3–5.

Except for generation of **20**, the other most probable oxidation at carbon C-14,15 in the reaction scheme (Figure 14) can occur in the case of 2,17-bidecarboxy-betanin **2** and 2,15-bidecarboxy-betanin **11** and is based on an alternative metal cation catalyzed oxidation mechanism in betacyanins [66]. This way, the 15- or 17-decarboxylation in the hydrogenated pyridinic ring of **2** or **11**, respectively, with concurrent 14,15-dehydrogenation should result in generation of 2,15,17-tridecarboxy-neobetanin **14** which is detected only at pH 3 (Figure 7). Subsequent oxidation of **14** at carbon C-2,3 completes this reaction pathway with the generation of 2,15,17-tridecarboxy-xanneobetanin **17**.

Detection of only minute quantities of decarboxylated derivatives, 15,17-bidecarboxy-betanin **3** and 2,15,17-tridecarboxy-betanin **13** should also be acknowledged, indicating that the 15,17-decarboxy path of betanin degradation is negligible (Figure 7 and Figure 8).

## 3. Materials and Methods

### 3.1. Reagents

Citric acid, Formic acid, acetic acid, EDTA, LC-MS grade methanol and water, and HPLC grade acetone were obtained from Sigma Chemical Co. (St. Louis, MO, USA).

### 3.2. Experiments on the Influence of Citrates and EDTA on the BRE Extract

The influence of citrates and EDTA on stability of the heated BRE extract obtained from FutureCeuticals, Inc. (Momence, IL, USA) [65] and generation of various betanin derivatives was performed according to a previously published procedure with modifications associated with the addition of reagents [71]. The extract aqueous stock solution (50 mL) was prepared at a concentration of 0.75 g/L and was 10× diluted in microplate wells up to 200 μL. Each well contained 20 μL of acetate/phosphate or citrate buffers at pH 3–8 (20 mM) and selected wells also contained 20 μL saturated EDTA solution. These samples were heated at 30, 50 or 85 °C in a thermostat for a few days, hours or 1 h, respectively, monitored by spectrophotometry in a microplate reader Tecan Infinite 200 (Tecan Austria GmbH, Grödig/Salzburg, Austria). During the experiments, additional aliquots (20 μL) of the heated samples were taken for LC-DAD-ESI-MS/MS analyses after 20× dilution. All the experiments were performed in triplicate.

### 3.3. LC-DAD-ESI-MS/MS Analyses

Quantitative analyses of the tested pigment mixtures were performed on a mass spectrometric system (LCMS-8030, Shimadzu, Kyoto, Japan) coupled to LC-20ADXR HPLC pumps, an injector model SIL-20ACXR, and a photo diode array detector (PDA) model SPD-M20A. The units were controlled with LabSolutions software, version 5.60 SP1 (Schimadzu). The separation was performed at 40 °C on a Kinetex C18 (150 mm × 4.6 mm i.d., 5.0 μm) chromatographic column preceded by a guard column of the same material (Phenomenex, Torrance, CA, USA). The flow rate was 0.5 mL/min and the injection volume was 10 μL.

Determined compounds were separated under the following elution gradient system composed of 2% aqueous formic acid (A) and pure methanol (B): 0 min, 10% B; increasing linearly to 12 min, 40% B; increasing linearly to 15 min, 60% B; increasing linearly to 19 min, 90% B.

Optical detection was performed in the full PDA range and at selected wavelengths (440, 480, 505 and 540 nm). The ionization electrospray source operated in positive mode (ESI+) at an electrospray voltage of 4.5 kV, capillary temperature at 250 °C and using N_2_ as a sheath gas. The LC-MS system was controlled with the LabSolutions software recording total ion chromatograms, mass spectra, ion chromatograms in selected ion monitoring mode (SIM) as well as the fragmentation spectra. Argon was used as the collision gas for the collision-induced dissociation (CID) experiments. The relative collision energies for MS/MS analyses were set at −35 V.

## 4. Conclusions

Addition of citrates to beetroot betalain-rich extract (BRE) solutions has contradictory effects on betanin and neobetanin stability in comparison to acetate/phosphate buffers and depends on pH range. The highest stability of betacyanins from the beetroot betalain-rich extract (BRE) is observed at pH 5 in acetate/phosphate buffers which is more evident at 85 °C. The highest impact of EDTA addition on the pigment retention in acetate buffers is observed at pH 3–5 and 8 at temp. 85 °C. At lower temperatures, pigment stability in more acidic conditions is still at higher levels even without the addition of citrates or EDTA. This demonstrates that applying low pH is much more destructive for the pigments at higher temperatures. In comparison to acetic buffers without EDTA, addition of citric buffers at 85 °C results in a significant stabilizing effect of the substrate pigments at a retention of 17–24% (betanin/isobetanin and neobetanin) at pH 3–4 (compared to 3–6% in acetic buffers) but increases their reactivity at pH 6–8 which is reflected in a lowered retention level (16–18% and 1–3% at pH 6 and 8, respectively). A similar tendency is observed at 30 and 50 °C for pH 7–8.

The most striking effect on generation of betanin derivatives during heating is 2-decarboxylation which preferentially proceeds in the most acidic environment and this generation rate at 85 °C is much higher in the citrate buffers compared to acetates. This results in the generation of 2-decarboxy-betanin from betanin but also 2-decarboxy-neobetanin from neobetanin. In the next reaction steps, from the intermediary mono-decarboxylated betanins, the formation of two derivatives, 2,17-bidecarboxy-betanin/-isobetanin and 2,15-bidecarboxy-betanin follows a similar trend, especially at 85 °C, where the acidic environment is preferential. The citrates enhance their formation at 30 and 50 °C.

## Figures and Tables

**Table 1:** Chromatographic, spectrophotometric, and mass spectrometric data of detected betanin-based decarboxylated and dehydrogenated derivatives in the betalain-rich extract (BRE) heated in acetate/phosphate and citrate buffers at 30, 50 and 85 °C.

			t_R_	λ_max_	*m/z*
No.	Pigment	Abbreviation	[min]	[nm]	[M + H]^+^
	*non-decarboxylated betacyanins*				
**1/1′**	betanin/isobetanin	Bt/IBt	6.6/7.3	536	551
**10**	neobetanin	NBt	10.3	468	549
	*mono-decarboxylated betacyanins*				
**2/2′**	17-decarboxy-betanin/-isobetanin	17-dBt/IBt	7.1/7.7	505	507
**4**	15-decarboxy-betanin	15-dBt	8.3	527	507
**5/5′**	2-decarboxy-betanin/-isobetanin	2-dBt/IBt	8.9	533	507
	*bi- and tri-decarboxylated betacyanins*				
**3**	15,17-bidecarboxy-betanin ^a^	15,17-dBt	8.3	494	463
**7/7′**	2,17-bidecarboxy-betanin/-isobetanin	2,17-dBt/IBt	9.6	507	463
**11**	2,15-bidecarboxy-betanin	2,15-dBt	10.4	532	463
**13**	2,15,17-tridecarboxy-betanin ^a^	2,15,17-dBt	10.7	503	419
	*mono-decarboxylated dehydro-betacyanins*				
**8**	2-decarboxy-xanbetanin ^a^	2-dXBt	9.6	446	505
**18**	2-decarboxy-neobetanin	2-dNBt	12.0	480	505
**20**	2-decarboxy-xanneobetanin	2-dXNBt	12.7	422	503
	*bi-decarboxylated dehydro-betacyanins*				
**6**	2,17-decarboxy-xanbetanin ^a^	2,17-dXBt	9.5	460	461
**12**	2,15-bidecarboxy-xanbetanin ^a^	2,15-dXBt	10.6	478	461
**16**	2,17-bidecarboxy-neobetanin ^a^	2,17-dNBt	11.7	459	461
	*bi-decarboxylated xanneobetacyanins*				
**15**	2,17-bidecarboxy-xanneobetanin	2,17-dXNBt	11.4	407	459
**19**	2,15-bidecarboxy-xanneobetanin ^a^	2,15-dXNBt	12.3	427	459
	*tri-decarboxylated dehydro-betacyanins*				
**9**	2,15,17-tridecarboxy-xanbetanin ^a^	2,15,17-dXBt	9.9	-	417
**14**	2,15,17-tridecarboxy-neobetanin ^a^	2,15,17-dNBt	11.3	442	417
**17**	2,15,17-tridecarboxy-xanneobetanin	2,15,17-dXNBt	11.9	394	415

^a^ Tentatively identified.

## Data Availability

Not applicable.
